# Supplementary material for: Charge-density wave mediated quasi-one-dimensional Kondo lattice in stripe-phase monolayer 1T-NbSe2
Source: Nat Commun. 2024 Feb 3;15:1039. doi: 10.1038/s41467-024-45335-7 (PMC10838322; doi:10.1038/s41467-024-45335-7)
Supplement: Supplementary file 1 — Supplementary Information [file 41467_2024_45335_MOESM1_ESM.pdf]

Supplementary Information for

Charge-density Wave Mediated Quasi-one-dimensional Kondo Lattice in Stripe-phase Monolayer 1T-$NbSe_2$

Zhen-Yu Liu[1&], Heng Jin[2,3&], Yao Zhang[1&], Kai Fan[1&], Ting-Fei Guo[1], Hao-Jun Qin[1], Lan-Fang Zhu[1], Lian-Zhi Yang[1], Wen-Hao Zhang[1], Bing Huang[2,3#] and Ying-Shuang Fu[1,4*]

1. School of Physics and Wuhan National High Magnetic Field Center, Huazhong University of Science and Technology, Wuhan 430074, China
2. Department of Physics, Beijing Normal University, Beijing 100875, China
3. Beijing Computational Science Research Center, Beijing 100093, China
4. Wuhan Institute of Quantum Technology, Wuhan 430206, China

& These authors contribute equally to this work.

*yfu@hust.edu.cn

[#]Bing.Huang@csrc.ac.cn

Contents

1. Supplementary Note 1: Cotunneling model.
2. Supplementary Note 2: Influence of strain.
3. Supplementary Note 3: Exclusion scenarios of SDW and inhomogeneous CDW.
4. Supplementary Fig. 1: Reversible transition between 1T phase and stripe phase.
5. Supplementary Fig. 2: Morphology of 1T phase and stripe phase.
6. Supplementary Fig. 3: Se-deficient line defects in stripe phase.
7. Supplementary Fig. 4: Morphology and d*I*/d*V* spectra of wide stripes.
8. Supplementary Fig. 5: STM images of the narrow stripe.
9. Supplementary Fig. 6: d*I*/d*V* maps of the narrow stripe.
10. Supplementary Fig. 7: Magnetic field dependence of the anti-resonance state.
11. Supplementary Fig. 8: Spatial distribution and temperature dependences of the spectral peak.
12. Supplementary Fig. 9: Structure of narrow stripes.
13. Supplementary Fig. 10: Orital-projected density of states (DOS) of CDW phase and charge distribution at the Fermi level.
14. Supplementary Fig. 11: The influence of substrate on SD phase and stripe phase.
15. Supplementary Fig. 12: Magnetic configurations in the calculation of exchange interaction.
16. Supplementary Fig. 13: Comparison of band structure of $NbSe_2$ stripe-phase and SD-phase.
17. Supplementary Fig. 14: Spatial distribution of magnetic moments of $NbSe_2$ stripe-phase at different energy range.
18. Supplementary Fig. 15: Influence of defects to Kondo states.
19. Supplementary Fig. 16: The symmetric oscillation on both sides of the defects
20. Supplementary Fig. 17: Cotunneling model and the spectrum of simulations.
21. Supplementary Fig. 18: DFT-calculated local DOS with/without 3% tensile strain.
22. Supplementary Fig. 19: DFT-calculated two possible SDW states.

23. Supplementary Fig. 20: DFT-calculated momentum-dependent bare susceptibility $\chi_0(\boldsymbol{q})$ of spin-unpolarized $NbSe_2$ stripe-phase under different strain ε.

SUPPLEMENTARY NOTES

Supplementary Note 1: Cotunneling model.

To reproduce the observed variation of the spectral lineshape, we modeled the tunneling spectra with the cotunneling model that has been well-established for the Kondo lattice system [1][2]. This model is more complex than the simple Fano lineshape fitting, but is more precise to describe the Kondo lattice spectra. The modelling is based on a hole-like conduction band $\mathrm{E}_k^c = -2\mathrm{t}(\cos \mathrm{k}_x + \cos \mathrm{k}_y) + \mu$ (green band in Fig. S17a) and a heavy flat band $\mathrm{E}_k^f = -2\chi_0(\cos \mathrm{k}_x + \cos \mathrm{k}_y) + \varepsilon_0^f$ (red band in Fig. S17a) near the Fermi energy (t: nearest neighbor hopping of the conduction electrons; μ: chemical potential; χ_0: nearest site spin correlation; ε_0^f: the position of the heavy band respect to Fermi energy). The coherent Kondo screening gives two heavy fermion bands: $E_k^{\pm} = \frac{E_k^c + E_k^f}{2} + \sqrt{\left(\frac{E_k^c - E_k^f}{2}\right)^2 + \nu^2}$; where ν is the hybridization amplitude between the conduction and heavy flat bands. The differential conductance dI/dV is given by $dI/dV \propto \sum_{i,j=1}^{2}[tImG(k,\omega)]_{ij}$; where t depicts the tunneling ratio between the conduction band and the heavy flat band, and $G(k,\omega)$ is the full Green's function describing the hybridization between the above two bands, whose details are seen in Ref. [2].

Based on this model with the same band structure shown in Fig. S17a, we simulate three dI/dV spectra with three selected values of t_f/t_c of 0.15, 0.03 and 0.002, where t_f and t_c are the tunneling amplitudes to the heavy and light bands, respectively. The simulated spectra shown in Fig. S17b well reproduce the experimental spectra in Fig. S8d and Fig. 3, demonstrating those spectra are all from Kondo resonances, but with different tunneling ratio between the heavy and electron bands, presumably due to the influence of strain.

Supplementary Note 2: Influence of strain.

Besides, to examine the strain effect, DFT calculations are performed to unveil the redistribution of tunneling channels under strain. The tunneling spectra are simulated by calculating local DOS (LDOS) in spheres ~ 4Å above Nb atoms, as shown in Fig. S18. We note that in our DFT

simulation the one-particle mean-field approach could not take the physics of paired Kondo singlet into account. However, the qualitative analysis could also provide insights into the large variation of tunneling channels of the stripe-phase monolayer $NbSe_2$. The strength of local moments at ~ 0.5 eV below the Fermi level hasn't been influenced strongly (red lines) by the strain.-However, strain strongly affects the LDOS of conduction electrons (blue lines) at the shifted Fermi level. For example, by applying 3% tensile strain, the LDOS is strongly suppressed at the shifted Fermi level (only ~1/3 of the unstrained one). This indicates the tunneling from the tip to the conduction electrons could also be strongly suppressed. Overall, the conduction electrons can vary strongly when strain is applied.

Supplementary Note 3: Exclusion scenarios of SDW and inhomogeneous CDW.

Here we evaluate whether the narrow spectral features at the Fermi level could be caused by other scenarios such as spin density wave (SDW) or charge density wave (CDW) caused by inhomogeneities. While the Kondo state cannot be reproduced from first-principles calculations, the SDW state should in principle be captured from first-principles calculations. Although without knowing the period of SDW and arrangement of moments we could not exclude all SDW possibilities, we inspect two possible SDWs from first-principles calculations. As shown in Fig. S19(a), we see no obvious DOS reduction at the Fermi level for SDW1 but a strong DOS reduction for SDW2. However, no dips or peaks ~ several mV can be observed in the shifted Fermi level, indicating the small dips and peaks around the Fermi level found in experiments may not be attributed to SDWs. It is also worth noting that the momentum-dependent susceptibility can guide to finding potential SDW orders. Although the calculation of interacting susceptibility requires many-body perturbation theory, which is beyond the scope of our manuscript, the non-interacting (bare) susceptibility under different strains has been calculated based on the formula $\chi_0(q) = \frac{1}{N}\sum_k \frac{f(\varepsilon_k) - f(\varepsilon_{k+q})}{\varepsilon_{k+q} - \varepsilon_k}$ with $\varepsilon(\boldsymbol{k})$ being the DFT-calculated eigenstates of spin-unpolarized $NbSe_2$ stripe-phase and $f(\varepsilon)$ being the Fermi-Dirac distribution. The results, as plotted in Fig. S20, clearly show that no peak can be found at a certain $\boldsymbol{q}$ and the strain does not influence the susceptibility much.

Moreover, these prominent variations in low energy range shown in Figure. S8 are not caused

by the coupling of CDW with inhomogeneities. As discussed in Ref. [3], the inhomogeneities will produce a pair of in-gap states that are symmetrical with respect to the center of the CDW gap, and they are typically located at the CDW gap edges. In our experiments, the CDW gap center is located at -0.1 V, while the low-energy features we observed in Fig. S8 are located near the Fermi surface, which are not symmetrical with respect to the CDW gap center. Therefore, the low-energy characteristics we observe are distinct from in-gap states induced by inhomogeneities. Further, as observed in Fig. S8, the modified low-energy features that are affected by local perturbations can be reproduced with the cotunneling model by changing the ratio of the two tunneling paths (Fig. S17), which confirms that the low-energy features are specifically Kondo resonances.

SUPPLEMENTARY FIGURES

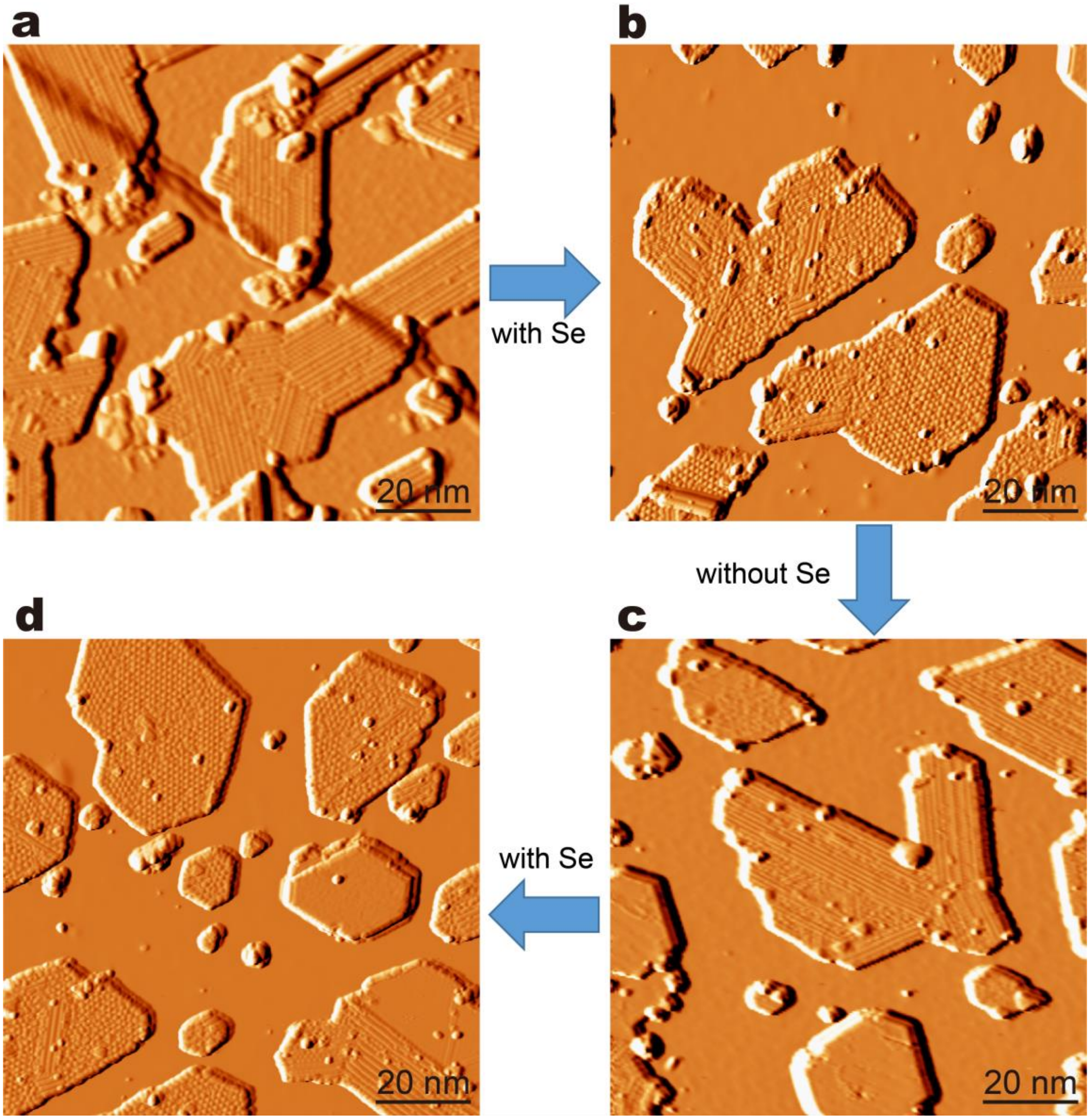

Supplementary Fig. 1 Reversible transition between 1T phase and stripe phase. a, STM image of monolayer stripe phase $NbSe_2$ on graphene. **b,** STM image from the same sample in **a** after annealing at 600 °C for 60 min with Se flux. The stripe phase $NbSe_2$ transforms into 1T-$NbSe_2$, showing the star of David superstructure. **c,** STM image from the same sample in **b** after annealing at 600 °C for 60 min without Se flux. Except for a few smooth-surfaced islands, namely 1H-$NbSe_2$, which remained unchanged, 1T-$NbSe_2$ re-transforms into stripe phase $NbSe_2$. **d,** STM image from the same sample in **c** after annealing at 600 °C for 60 min with Se flux. All STM images were acquired at condition V_b = -1.0 V and I_t = 20 pA, and displayed in derivative mode.

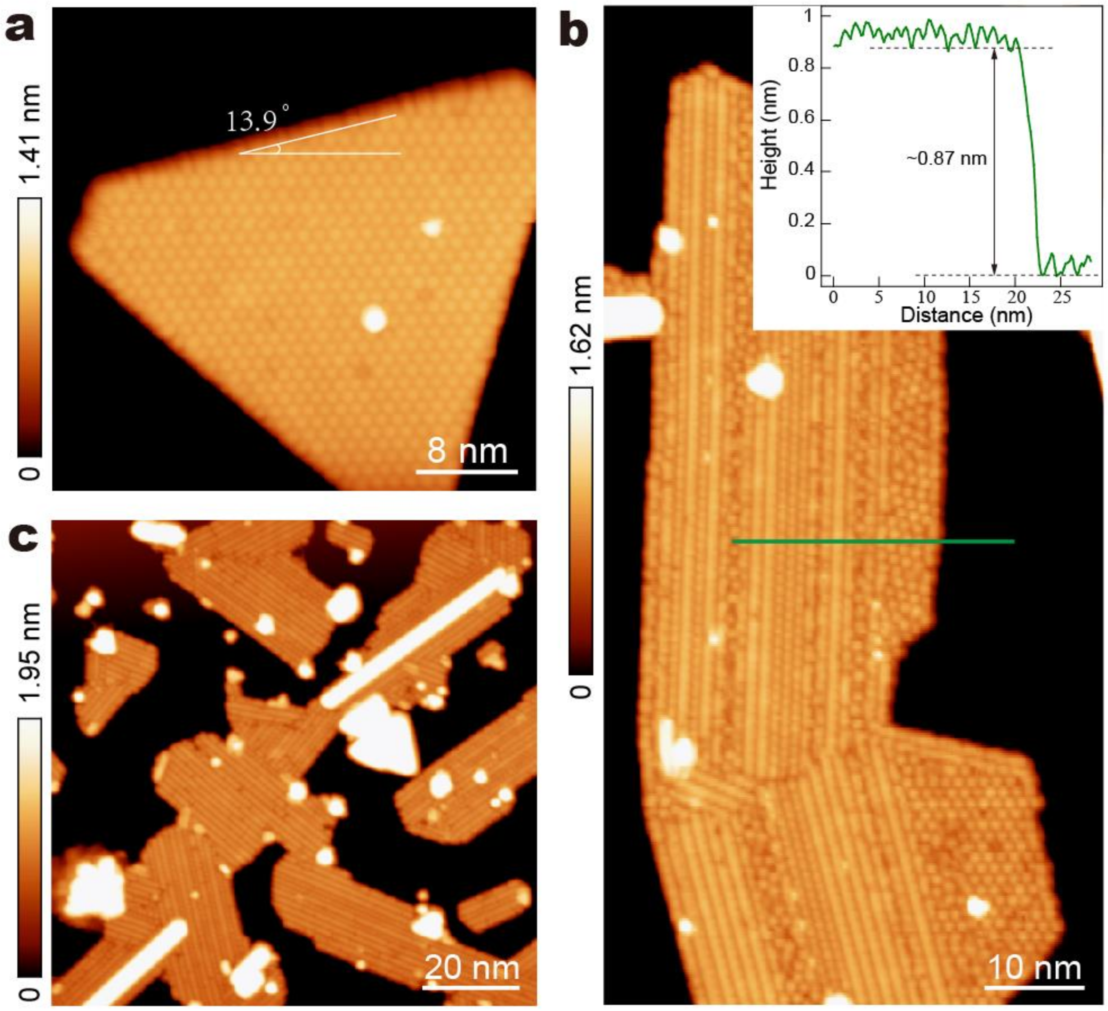

Supplementary Fig. 2 Morphology of 1T phase and stripe phase. a, STM image of 1T-$NbSe_2$ grown with high Nb:Se flux ratio. **b,** STM image of an island with two coexisting phases grown with medium Nb:Se flux ratio. The inset shows a height profile along a green line, indicating that the stripe phase has the same layer height as the 1T phase. **c,** STM image of stripe phase $NbSe_2$ acquired by annealing the sample shown in **b** at 600 °C for 30 min. All STM images were acquired at condition V_b = -1.0 V and I_t = 20 pA.

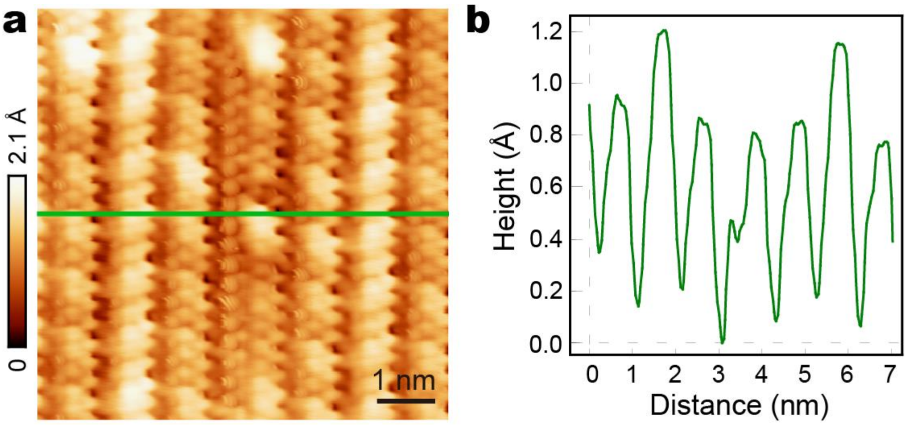

Supplementary Fig. 3 Se-deficient line defects in stripe phase. a, Atomic resolution STM image of the stripe phase (V_b = 20 mV and I_t = 3 nA). **b,** Height profile along a green line in **a**.

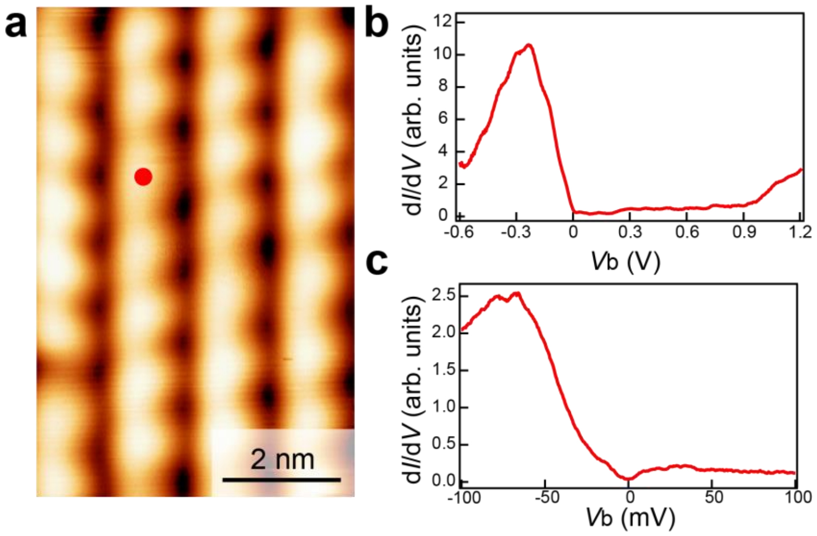

Supplementary Fig. 4 Morphology and d*I*/d*V* spectra of wide stripes. a, STM image (V_b = -0.6 V and I_t = 10 pA) of the wide stripe. **b, c**, d*I*/d*V* spectra with different energy range taken at the location marked with red dot in **a**. No Kondo spectrum is observed from **c**.

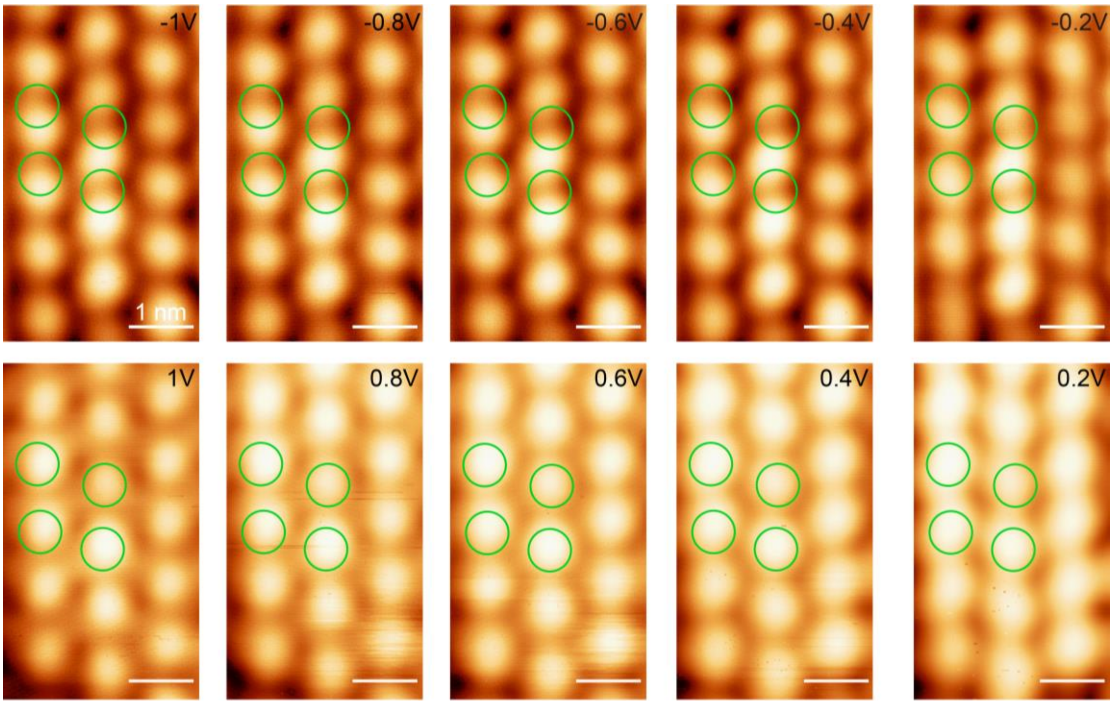

Supplementary Fig. 5 STM images of the narrow stripe. The STM images at different bias voltages (tunneling current is 30 pA). The negative bias topography is shifted by half a phase along the stripe direction relative to the positive bias topography. The green circle marks the same location for all images.

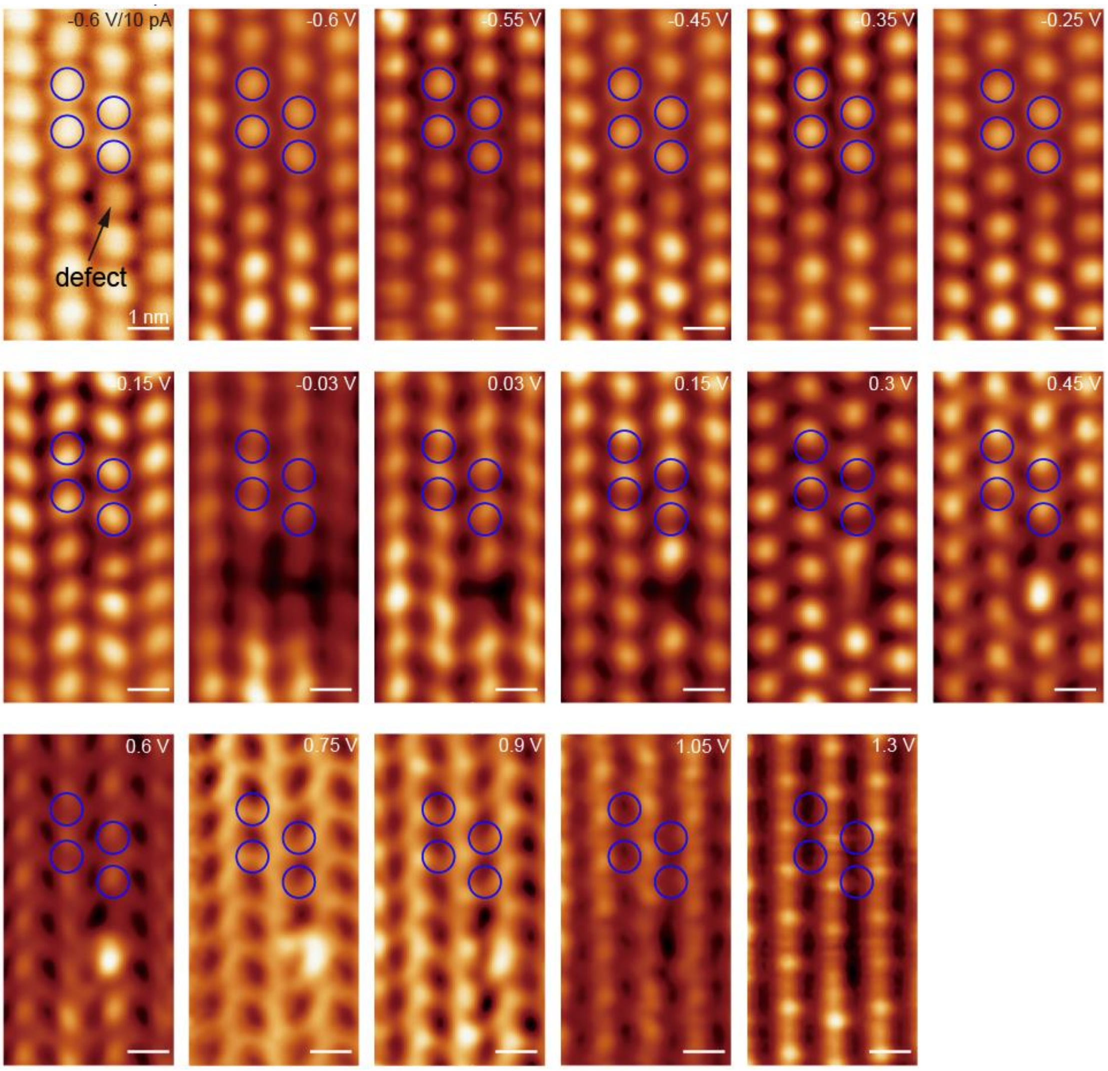

Supplementary Fig. 6 d*I*/d*V* maps of the narrow stripe. The first image is the STM topography of the narrow stripes, and the other images are the constant-height d*I*/d*V* maps at different bias voltages in the same area (for 0.03 V and -0.03 V, V_{mod} = 5 mV, the others V_{mod} = 20 mV). The blue circle marks the same location form all images.

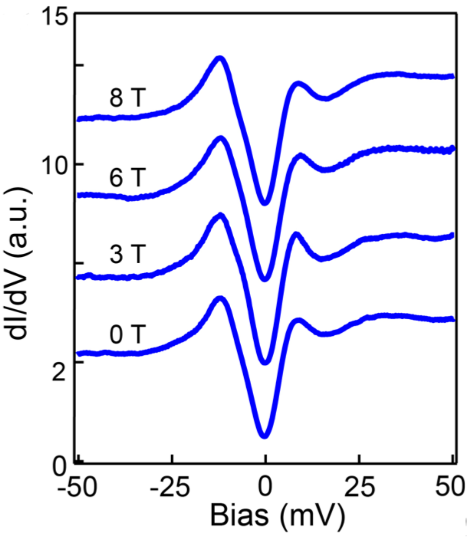

Supplementary Fig. 7 Magnetic field dependence of the anti-resonance state. d*I*/d*V* spectra measured at different external magnetic fields on the same location of the stripe-phase $NbSe_2$ (V_b = -50 mV, I_t = 300 pA, V_{mod} = 0.5 mV). The spectra are vertically offset for clarity. The Kondo resonance is not split at 8 T because its Zeeman splitting energy is way smaller than its Kondo peak width.

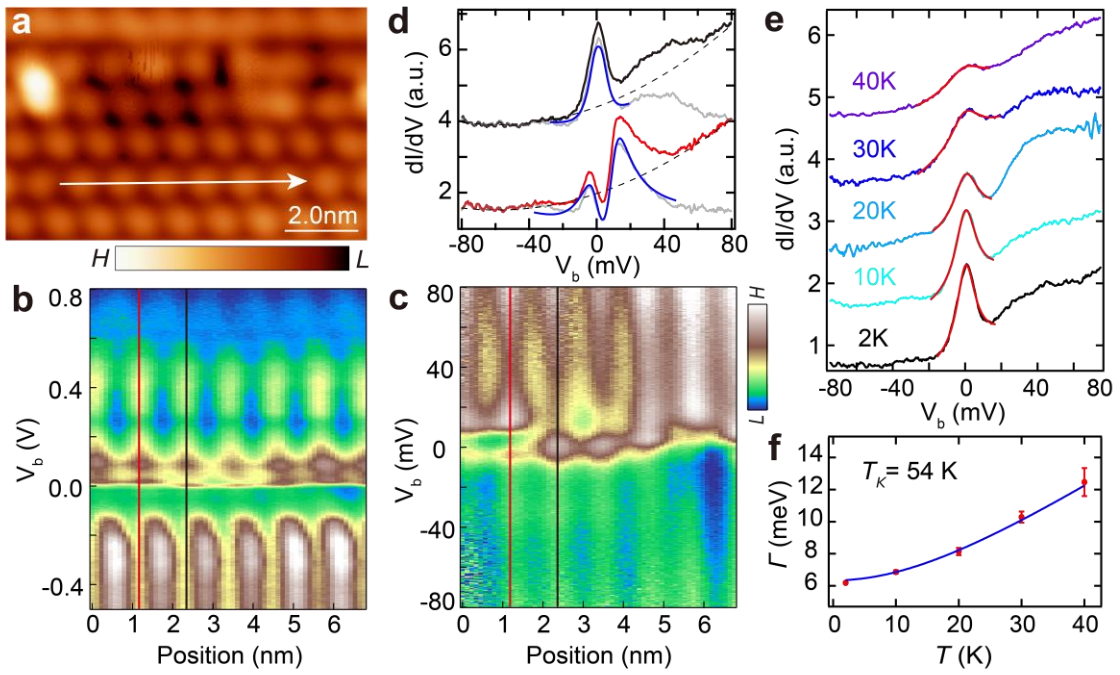

Supplementary Fig. 8 Spatial distribution and temperature dependences of the spectral peak. **a,** STM image (V_b = -0.5 V and I_t = 10 pA) of the stripe-phase $NbSe_2$ containing local inhomogeneity at the top region of the image. **b, c,** 2D conductance plot of different energy ranges taken along the white arrow in **a**. While the line spectra at large bias range of [-0.5, 0.8 eV] are relatively uniform with well-formed CDW state, the low energy range of [-80, 80 meV] indicates prominent variations, reflecting the strong impact of local perturbation, presumably strain effect, to the q factor. **d,** dI/dV spectra (V_b = -80 mV, I_t = 150 pA, V_{mod} = 1 mV) extracted from **c** marked with corresponding color lines, showing a narrow Kondo peak (black curve) and a Kondo gap with two enhanced peaks at the gap edges (red curve). Those spectra could be reproduced well after subtracting the background (grey curves) by the cotunneling model (blue curves) with parameters $t_f/t_c = 0.025$, $\gamma_f = 0.027\text{t}$ for the Kondo peak, and $t_f/t_c = -0.01, \gamma_f = 0.021\text{t}$ for the Kondo dip. The dashed lines are the fitted background spectrum from the red curve. Note that our applied magnetic field cannot split the Kondo peak, because of the larger Kondo peak width than the Zeeman splitting energy. **e,** Temperature evolution of the dI/dV spectra measured at the same location of the stripe-phase $NbSe_2$ (V_b = 80 mV, I_t = 200 pA, V_{mod} = 1 mV). Red curves are Fano fittings to the spectra. **f,** Extracted Kondo peak width Γ (red dots) against temperature. The blue curve is a fitting to the data according to the Kondo model, yielding a Kondo temperature of $T_k \approx$ 54 K.

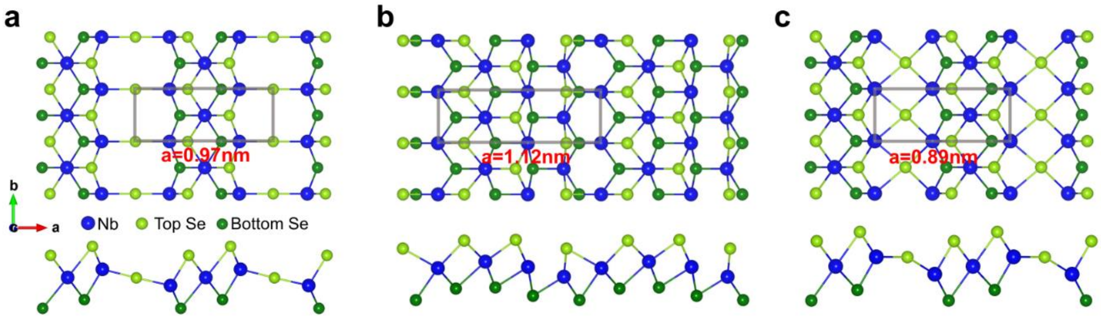

Supplementary Fig. 9 Structure of narrow stripes. a,b Narrow stripe structure of $NbSe_2$ referred to that in VSe_2 from different references [4][5]. **c,** Narrow stripe structure of $NbSe_2$ obtained by geometry optimization of structure in **a.** The primitive cell is illustrated with gray rectangles. The total energy of the structural model in **c** is ~ 0.11eV/atom lower than that in **a**.

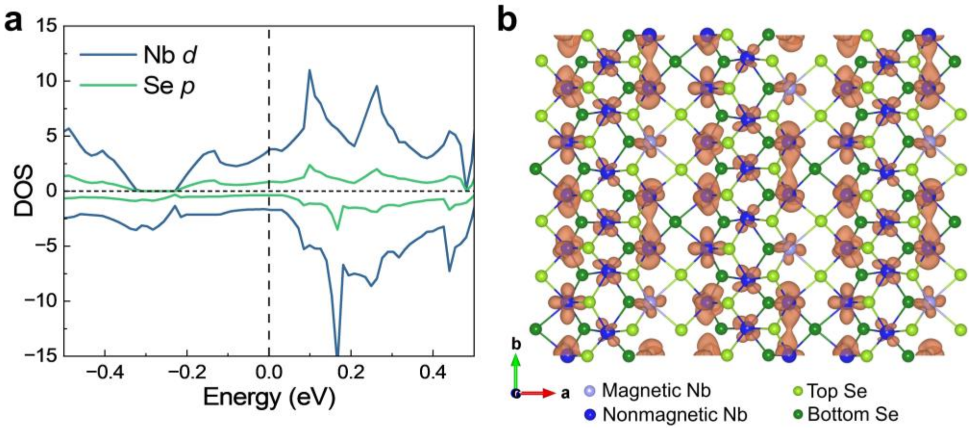

Supplementary Fig. 10 Orital-projected density of states (DOS) of CDW phase and charge distribution at the Fermi level. a, Orbital-projected DOS of $NbSe_2$. **b,** Charge distribution at the Fermi level with iso-surface ~1×10^{-4}e/Å^3. Nb atoms with (without) local magnetic moments are illustrated with shallow (deep) blue.

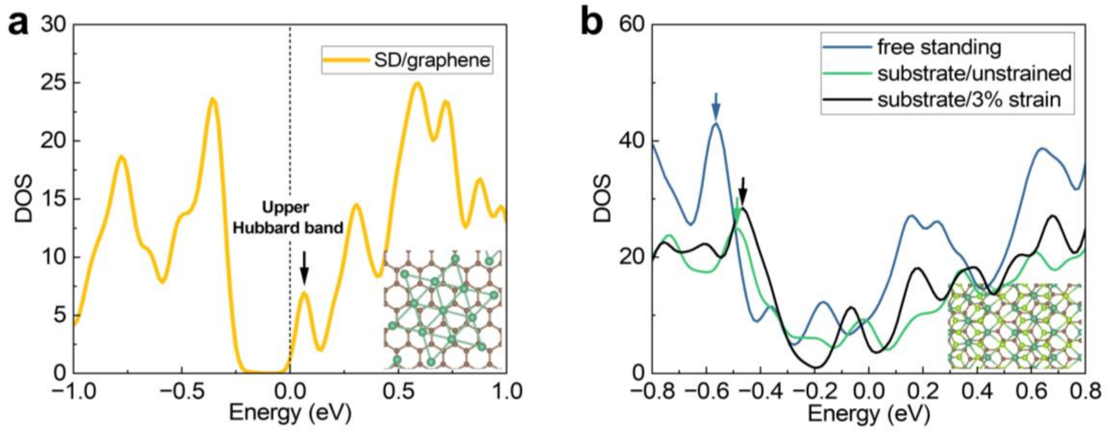

Supplementary Fig. 11 The influence of substrate on SD phase and stripe phase. a, Substrate-induced charge transfer effect on SD-phase $NbSe_2$. **b,** Joint strain- and substrate-induced energy shifts in stripe-phase $NbSe_2$.

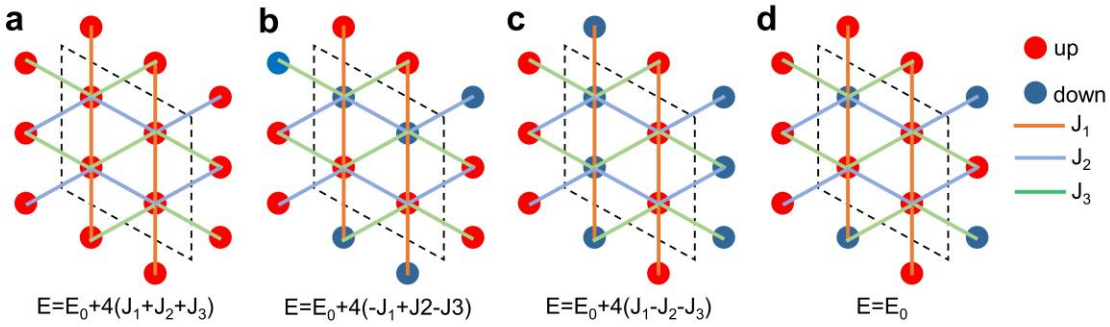

Supplementary Fig. 12 Magnetic configurations in the calculation of exchange interaction. a, Ferromagnetic, **b** and **c,** antiferromagnetic and **d** ferrimagnetic configurations. Red and blue dots indicate different spins on Nb atoms with major magnetic moments in the CDW structure (see Fig. 4c). The exchange interactions based on the Heisenberg model are illustrated with lines with different colors. Supercells to accommodate these magnetic configurations are shown with dashed lines. We note that for metallic systems, it may be less accurate to estimate the magnetic exchange interaction parameters using the Heisenberg model [6, 7], even the magnetic moments are localized, but the obtained J_1, J_2, J_3 values are still comparable.

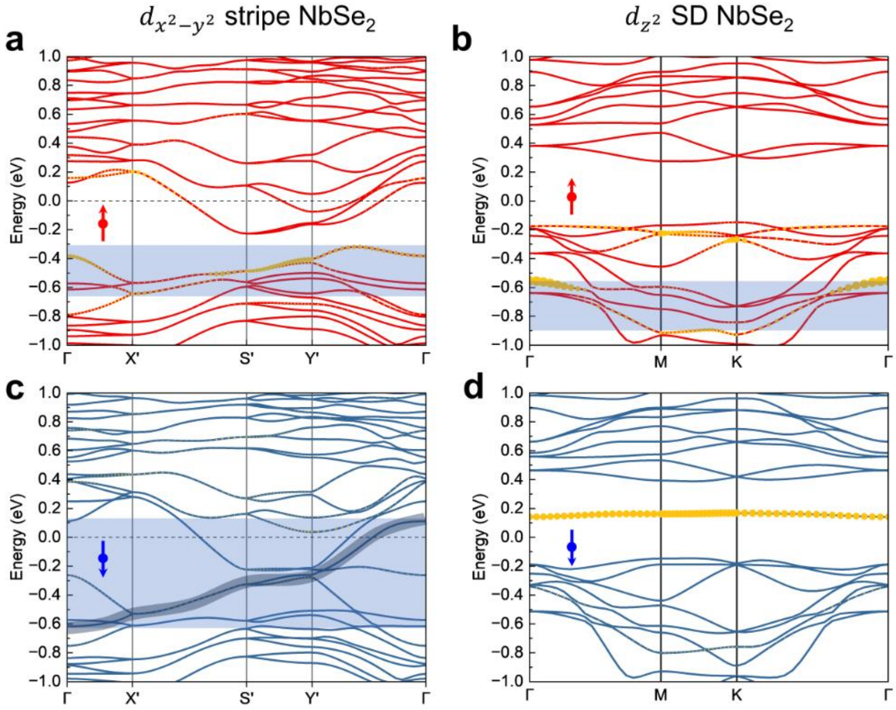

Supplementary Fig. 13 Comparison of band structure of $NbSe_2$ stripe-phase and SD-phase. a, Spin-up and **c,** spin-down bands of stripe-phase. **b,** Spin-up and **d,** spin-down bands of SD-phase. Orbitals mainly contributing to the magnetic moments ($d_{x^2-y^2}$ for stripe-phase and d_{z^2} for SD-phase) are projected and plotted with yellow dots. In **c** the thickened line indicates possible dispersive band coupled to local moments. The indication of bandwidth is shown with shadowed window.

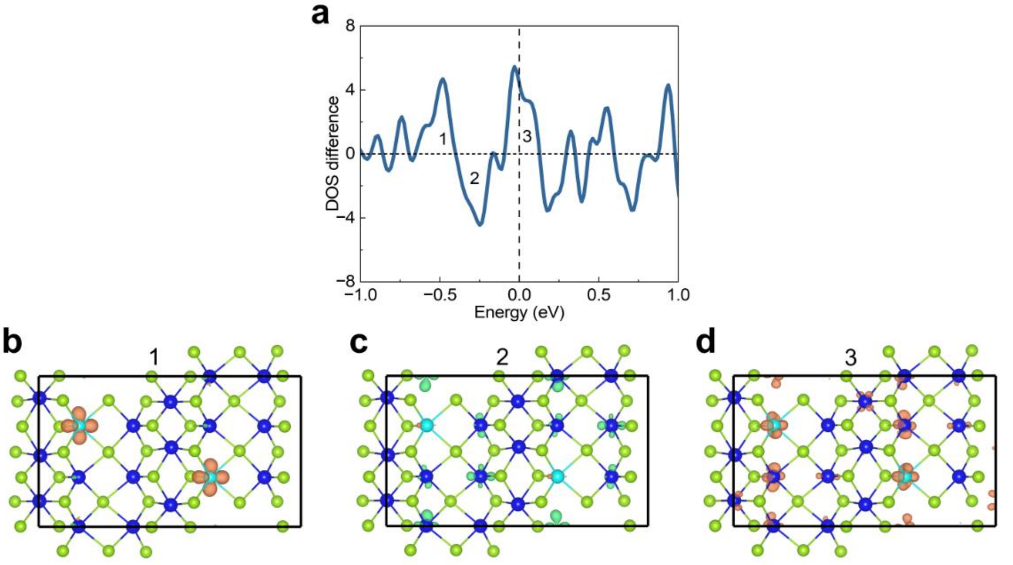

Supplementary Fig. 14 Spatial distribution of magnetic moments of $NbSe_2$ stripe-phase at different energy range. a, DOS difference (spin ↑ - spin ↓). **b**, **c**, and **d,** Magnetic moments at corresponding energy range 1(-0.65~-0.4 eV), 2(-0.4~-0.15 eV), and 3(-0.1~0.12 eV). The isosurface is set to 1×10^{-3} e/Å^3 and orange (blue) isosurface represents spin ↑ (spin ↓) magnetic moments. The Nb atoms proposed to provide local moments for the Kondo lattice are illustrated with shallow blue.

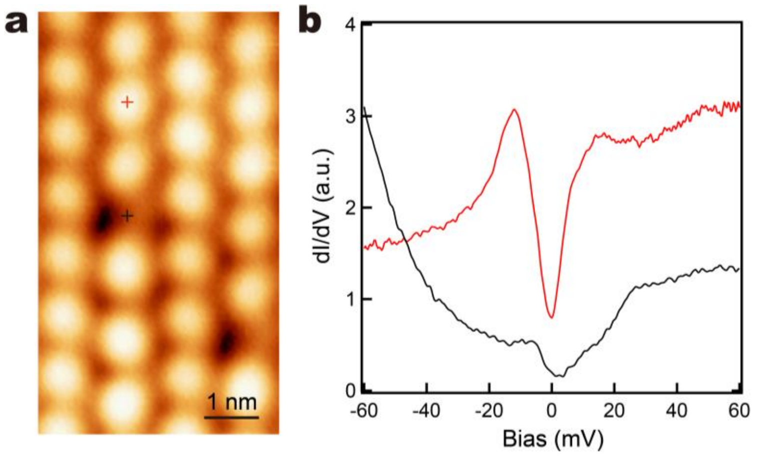

Supplementary Fig. 15 Influence of defects to Kondo states. a, STM image of narrow stripes with a defect (marked with black cross) (V_b = -1 V and I_t = 10 pA). **b,** dI/dV spectrum taken at corresponding colored crosses in **a** (V_b = -0.6 V, I_t = 100 pA, V_{mod} = 1 mV).

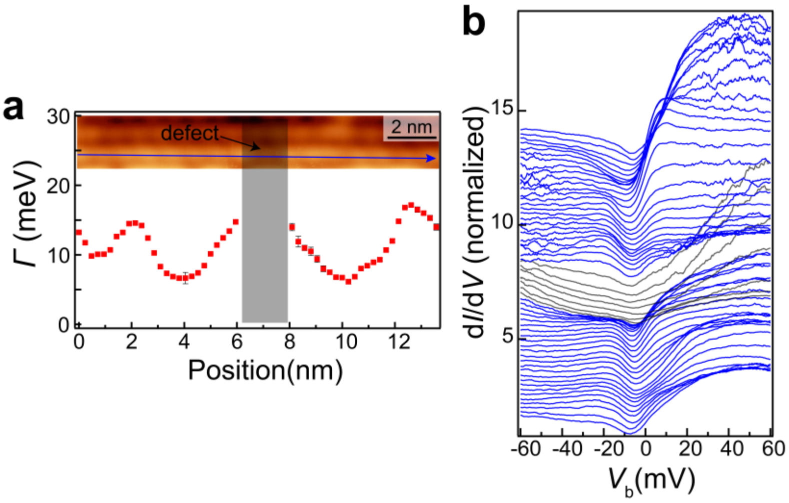

Supplementary Fig. 16 The symmetric oscillation on both sides of the defects. Spatial oscillation of the Kondo peak width *Γ* (red dots) cross defects along the strip direction, that are extracted by fitting the d*I*/d*V* spectra in **b**. Inset STM images show the stripe phase $NbSe_2$ with marked defects (black arrow) and the spectral locations (blue arrow). **b**, The d*I*/d*V* spectra taken along the blue arrow in the inset images of **a**, the grey curves are dI/dV spectra taken at the defect.

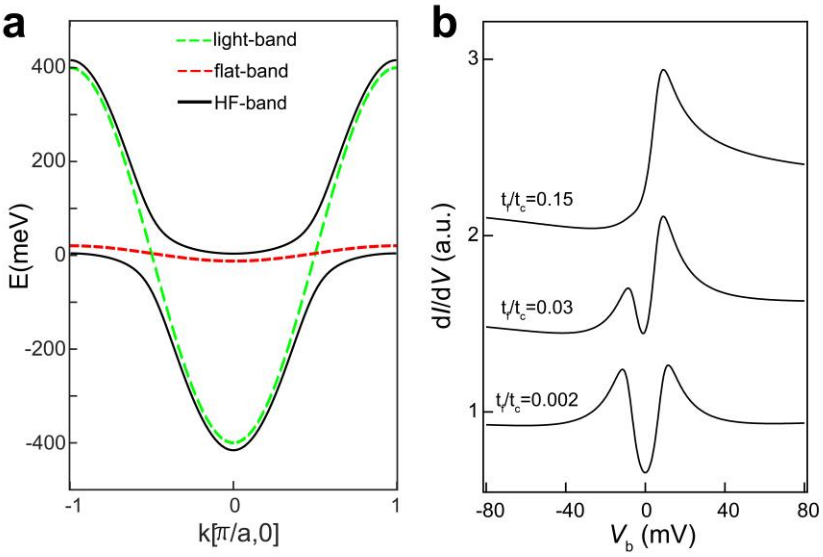

Supplementary Fig. 17 Cotunneling model and the spectrum of simulations. a, Dispersion of the conduction light (dashed green) and flat (dashed red) electronic bands and the hybridized heavy fermion bands (solid black) computed for $\mathrm{t} = 200\mathrm{meV}, \mu = 2\mathrm{t}, \chi_0 = 0.05t, \varepsilon_0^f = 0.1\mathrm{t}, \nu = 0.5\mathrm{t}, \gamma_f = 0.02\mathrm{t}$. **b**, Differential conductance computed using the same band structure parameters as in **a** for selected values of t_f/t_c. The value of ν is 0.5t for the top and middle curve, and 0.52t

for the bottom curve.

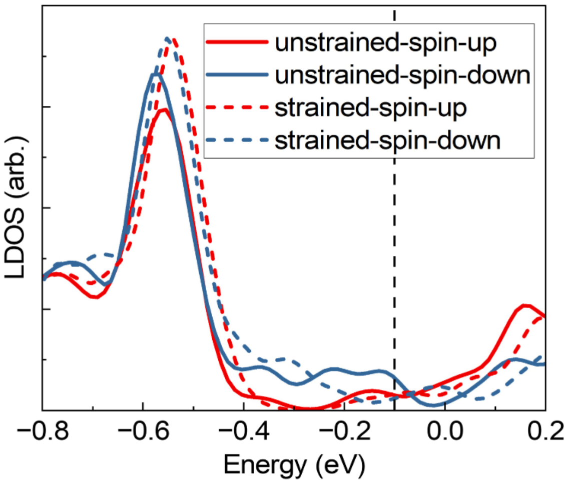

Supplementary Fig. 18 DFT-calculated local DOS with/without 3% tensile strain. The spin-↑ LDOS is calculated in a sphere above Nb atoms with local magnetic moments (shallow blue sites in Fig. S14). The spin-↓ LDOS is calculated in a sphere above Nb atoms neighboring to shallow blue Nb atoms, which provide conduction electrons at the shifted Fermi level (dashed line).

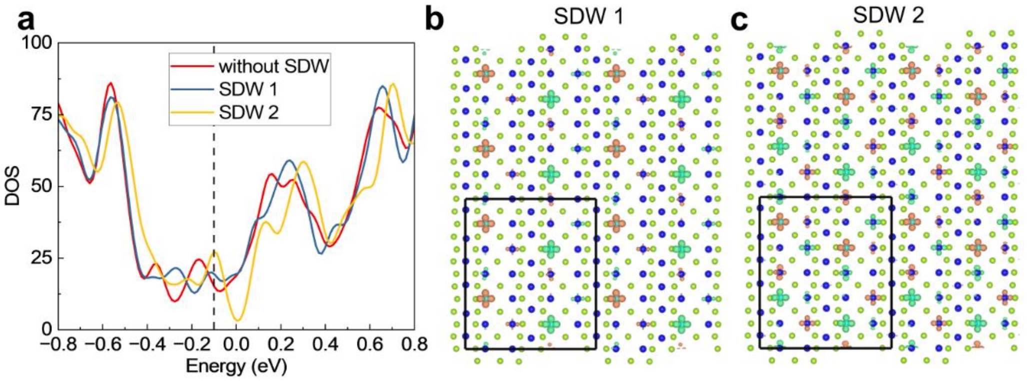

Supplementary Fig. 19 DFT-calculated two possible SDW states. a, DOS of SDW states compared with the original CDW state. The Fermi level is set to zero and the dashed line indicate the shifted Fermi level due to substrate. **b** and **c**, Two possible SDWs in the $NbSe_2$ stripe-phase. The local moments with spin ↑ (↓) are illustrated with orange (green) color.

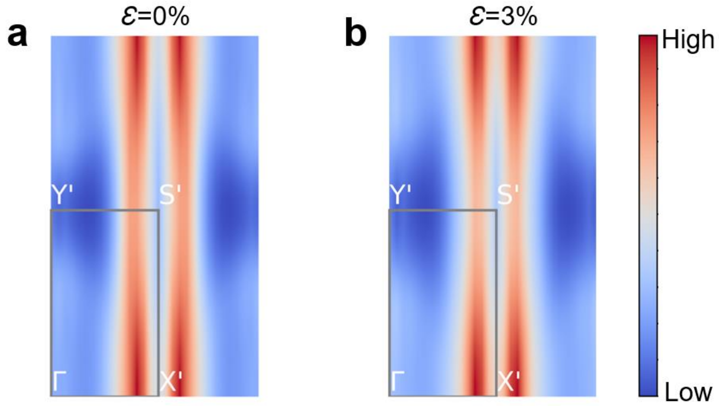

Supplementary Fig. 20 DFT-calculated momentum-dependent bare susceptibility $\chi_0(\boldsymbol{q})$ of spin-unpolarized $NbSe_2$ stripe-phase under different strain $\boldsymbol{\varepsilon}$. a, $\chi_0(\boldsymbol{q})$ without strain and **b**, $\chi_0(\boldsymbol{q})$ with 3% tensile strain.

Supplementary References:

[1] Maltseva, M., Dzero, M. & Coleman, P. Electron cotunneling into a Kondo lattice. Phys. Rev. Lett **103**, 206402 (2009).

[2] Aynajian, P., da Silva Neto, E., Gyenis, A. et al. Visualizing heavy fermions emerging in a quantum critical Kondo lattice. Nature **486**, 201–206 (2012).

[3] Tütto, I. & Zawadowski, A. Quantum theory of local perturbation of the charge-density wave by an impurity: Friedel oscillations. Phys. Rev. B **32**, 2449–2470 (1985).

[4] R. Chua, J. Yang, X. He, X. Yu, W. Yu, F. Bussolotti, P. K. J. Wong, K. P. Loh, M. B. H. Breese, K. E. Johnson Goh, Y. L. Huang, and A. T. S. Wee. Can Reconstructed Se-Deficient Line Defects in Monolayer VSe_2 Induce Magnetism?. Adv. Mater. **32**, 2000693 (2020).

[5] Liu Z L, Lei B, Zhu Z L, et al. Spontaneous formation of 1D pattern in monolayer VSe2 with dispersive adsorption of Pt atoms for HER catalysis. Nano Letters, **19**, 4897-4903 (2019).

[6] Yang B, Li Y, Xiang H J, Lin H Q, and Huang B, Moiré Magnetic Exchange Interactions in Twisted Magnets, Nature Comput. Sci. **3**, 314 (2023).

[7] Li Y, Xu S, Wang J, Wang C, Yang B, Lin H Q, Duan W H, and Huang B, Interplay between quantum anomalous Hall effect and magnetic skyrmions, PNAS 119, e2122952119 (2022).
